# MicroRNA-184 downregulates nuclear receptor corepressor 2 in mouse spermatogenesis

**DOI:** 10.1186/1471-213X-11-64

**Published:** 2011-10-24

**Authors:** Jingwen Wu, Jianqiang Bao, Li Wang, Yanqin Hu, Chen Xu

**Affiliations:** 1 Department of Histology & Embryology, Shanghai Jiaotong University School of Medicine, Shanghai 200025, China; 2 Shanghai Key Laboratory of Reproductive Medicine, Shanghai 200025, China

## Abstract

**Background:**

There have been increasing attentions on the role of small RNAs, especially microRNAs in post-transcriptional gene regulation during spermatogenesis. MicroRNA-184 (miR-184) has been shown to be mainly expressed in the testis and brain, and that its expression levels are by far the highest in the testis. However, the role of miR-184 in mammalian spermatogenesis remains unclear.

**Results:**

In this study, we demonstrated that miR-184 levels were increased during mouse postnatal testis development. Specifically, miR-184 expression was restricted to the germ cells from spermatogonia to round spermatids. Overexpression of miR-184 promoted the proliferation of a germ cell line, GC-1spg. Moreover, miR-184 downregulated *nuclear receptor corepressor 2 *(*Ncor2*) by targeting its 3' untranslated region through inhibiting NCOR2 protein translation.

**Conclusions:**

MiR-184 may be involved in the post-transcription regulation of mRNAs such as *Ncor2 *in mammalian spermatogenesis.

## Background

Spermatogenesis is a highly regulated process of germ cell differentiation that can be subdivided into three main phases: spermatogonial proliferation, meiosis of spermatocytes and spermiogenesis of haploid spermatids. The meiotic and haploid phases of spermatogenesis are characterized by high transcriptional activity but suppressed translational activity. Post-transcriptional control of gene expression in these phases is a significant feature of mammalian spermatogenesis [1]. MicroRNAs (miRNAs) are a family of small non-coding RNAs (typically 19~23 nt), which play important roles in regulating post-transcriptional gene silence through base-pair binding to their target mRNA [2]. Emerging evidences have suggested that the involvement of miRNAs in mammalian spermatogenesis. First, numerous miRNAs are exclusively or preferentially expressed in the mouse testis [3]. Second, the pattern of miRNA expression appears to be different between immature and mature mouse testis [4]. Last but not least, spermatogenesis is disrupted at the early stage of proliferation and/or early differentiation in mouse in which the *Dicer *gene, encoding an RNase III required for miRNA processing, has been deleted in the testis [5]. Additionally, several studies have indicated that some miRNAs participate in mammalian spermatogenesis. For example, miRNA-122a reduces the expression of the post-transcriptionally regulated germ cell *transition nuclear protein 2 *(*Tnp2*) mRNA in the mammalian testis [6]; miR-372 and miR-373 have been implicated as oncogenes in testicular germ cell tumors [7]; miR-383 is associated with male infertility and promotes testicular embryonal carcinoma cell proliferation by targeting *interferon regulatory factor-1*(*Irf1*) [8].

It has been shown that miR-184 is mainly expressed in the testis and brain, and that its expression levels in the testis are much higher than those in the brain [9,10]. However, the role of miR-184 in mammalian spermatogenesis remains unclear. Here, we reported for the first time that miR-184 expression levels increased with the postnatal development of the mouse testis and its expression was restricted to testicular germ cells. We further provided evidence that miR-184 could downregulate *nuclear receptor corepressor 2 *(*Ncor2*) by targeting its 3'- untranslated region (3'-UTR) and inhibiting NCOR2 protein translation. Our findings suggest that miR-184 may be involved in the post-transcription regulation of mRNAs such as *Ncor2 *in mammalian spermatogenesis.

## Results

### MiR-184 expression levels increased during the postnatal development of the mouse testis

**Figure 1:**
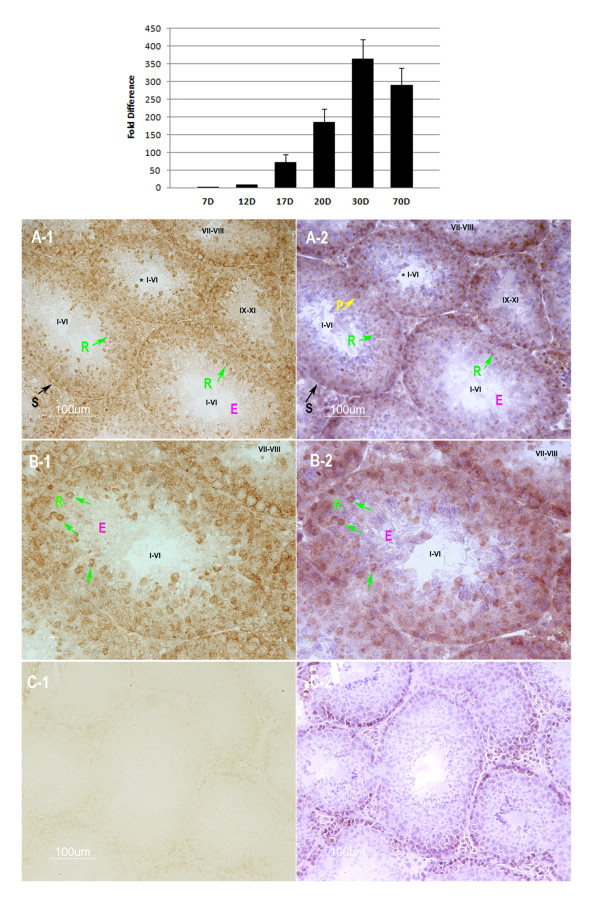
**Expression pattern of miR-184**. Upper panel, Relative quantity of miR-184 at different postnatal ages of mouse testes. *X axis*, different postnatal days of mouse testes; *Y axis*, miR-184 expression levels relative to postnatal day 7; Values are presented as the mean ± SD. Lower panel, Localization of miR-184 in adult mouse testes. MiR-184 (brown granules) was restricted to the cytoplasm of spermatogonia (S), spermatocytes (P) and round spermatids (R) in stage I-VI and stage VII-VIII, while the elongating or elongated spermatids (E) in stage IX-XI, stage I-VI were negative for miR-184. B-1 was a high magnification of the tubule in A-1 marked with (*). C-1 was the negative control using an LNA-modified, DIG-labeled scramble probe. Sections of A-1, B-1 and C-1 were counter-stained with hematoxylin as shown in A-2, B-2 and C-2. Roman numerals designate the stages of the seminiferous epithelium cycle: I-VI, stages I, II, III, IV, V or VI; VII-VIII, stages VII or VIII; IX-XI, stages IX, × or XI. Bars = 100 μm.

As shown in Figure 1, levels of miR-184 increased during the postnatal development of the mouse testis. At postnatal day 12 when zygotene spermatocytes appear [11], miR-184 levels increased by 9 fold compared with postnatal day 7. MiR-184 levels continued to accumulate thereafter with an increase by 71, 186, 362 and 289-fold at postnatal day 17, 20, 30 and 70, respectively.

### MiR-184 was located to the germ cells of mouse testis

The testis mainly contains two kinds of cell types: germ cells and somatic cells. Which cell type expresses miR-184 in the testis? To answer this question, *in-situ *hybridization assays were used to examine the miR-184 localization in the adult mouse testis. As shown in Figure 1, miR-184 was mainly detected in the cytoplasm of spermatogonia, spermatocytes in all stages of seminiferous epithelium. Round spermatids in stage I to stage VIII also expressed miR-184. While the elongating spermatids in stage IX and stage X, elongated and condensed spermatids in stage XI, stage I to stage VI did not show any positive signals. Moreover, Leydig cells in the interstitial tissue of the testis, peritubule myoid cells around the seminiferous tubule and Sertoli cells in the seminiferous tubule were negative for miR-184.

### MiR-184 overexpression promoted GC-1spg proliferation

**Figure 2:**
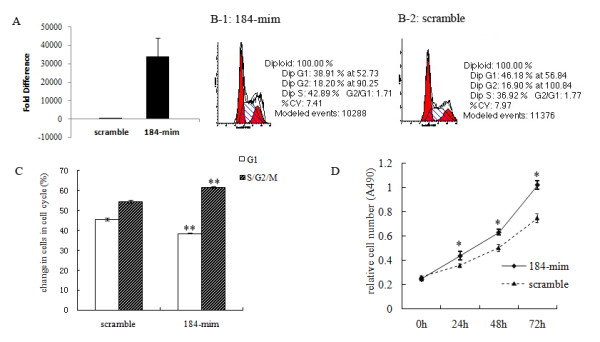
**MiR-184 promoted GC-1spg proliferation**. A, Quantitative RT-PCR showed the increased levels of miR-184 upon overexpression. B, Flow cytometry analysis of GC-1spg transfected with miR-184-mimic (B-1) or scrambled oligonucleotide (B-2). C, MiR-184 overexpression resulted in significantly lower number of cells in the G1 phase (*P *< 0.001) and significantly higher number of cells in the S/G/M2 phase (*P *< 0.001) compared with the negative control. D, Analysis of cell proliferation using MTS assay. *Y axis*, measurement of A490, reflecting the relative cell number. *X axis*, units of time in days. Measurements were taken at days 0 to 3 after transfection of GC-1spg with miR-184-mimic (solid line) or scrambled negative control (dash line). This experiment was repeated three times in triplicate. Values are presented as the mean ± SD. **P < 0.001, *P < 0.01.

To evaluate the characteristic of miR-184 in germ cells, we transfected a double-stranded RNA that mimics the miR-184 precursor (miR-184 mimic) into GC-1spg. GC-1spg is a kind of germ cell line that corresponds to a stage between type B spermatogonia and primary spermatocyte [12]. The cy3-labeled scramble oligonucleotide was used as a negative control. The high transfection efficiency was confirmed by observation red dots around nuclear under fluorescence light (additional file 1). The increased levels of miR-184 upon overexpression were demonstrated by quantitative RT-PCR (Figure 2A). FCS analysis revealed that miR-184 overexpression resulted in significantly lower number of cells in the G1 phase (*P *< 0.001) and significantly higher number of cells in the S/G/M2 phase (*P *< 0.001) compared with the scramble negative control (Figure 2 B, C). This result suggests that overexpression of miR-184 in GC-1spg could promote cells from G1 phase to S/G/M2 phase, indicating that miR-184 could induce GC-1spg proliferation. To further evaluate the effects of miR-184 on GC-1spg, MTS [3-(4, 5-dimethylthiazol-2-yl)-5-(3-carboxymethoxyphenyl)-2-(4-sulfophenyl)-2H-tetrazolium, inner salt; MTS] assays were used. MTS is a compound that is bio-reduced by living cells into a formazan product that is coloured and soluble in cell culture medium. The quantity of formazan product as measured by the amount of 490 nm absorbance is directly proportional to the number of living cells in culture. We measured the 490 nm absorbance at different time after transfection of miR-184 into GC-1spg. The MTS assay showed that the relative cell number was significantly increased with overexpression of miR-184 (*P *< 0.01) (Figure 2D). These results indicate that miR-184 promotes GC-1spg proliferation.

### MiR-184 targeted *Ncor2 *at its 3'UTR

**Figure 3:**
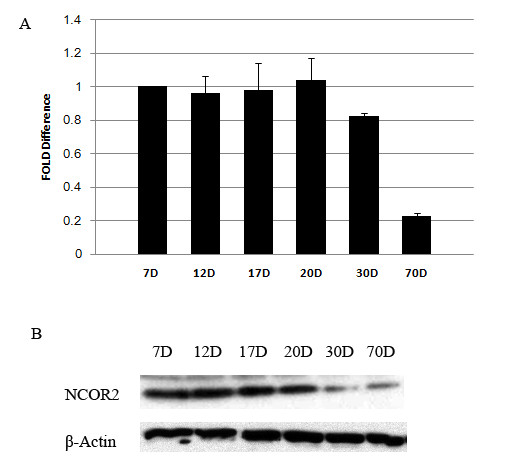
**Relative quantity of *Ncor2 *mRNA and NCOR2 protein at different postnatal ages of mouse testes**. A, Quantitative RT-PCR assay. *X axis*, different postnatal days of mouse testes; *Y axis*, *Ncor2 *expression levels relative to postnatal day 7; Values are presented as the mean ± SD. B, Western-blot assay. NCOR2 protein levels decreased from postnatal 30 day testis to adult mouse testis comparing with those in postnatal 7 day testis.

**Figure 4:**
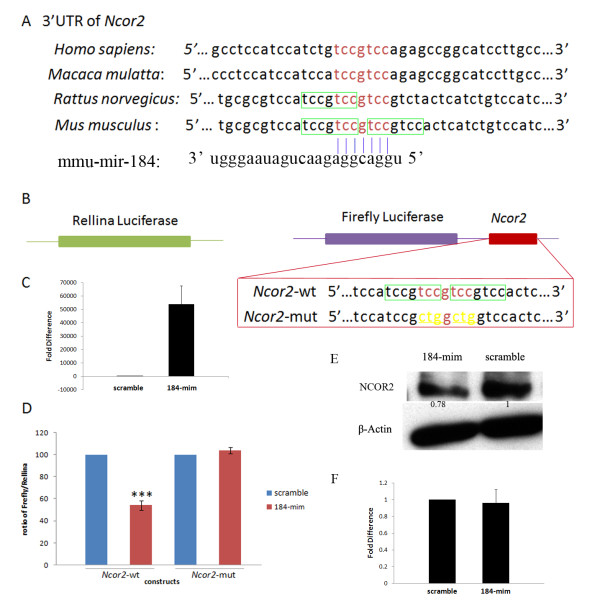
**MiR-184 targeted *Ncor2 *at its 3'UTR**. (A) Sequence alignment of the miR-184 complementary site in the 3'-UTRs of *Ncor2 *mRNAs. The region complementary to the miR-184 seed match highly conserved among human, Macaca mulatta, rat and mouse is in red. And the overlapping complementary region in rat and mouse to the miR-184 seed match is in red or boxed. (B) Constructs and mutated target sequence of *Ncor2*. A 517 bp region of the 3'-UTR of mouse *Ncor2 *containing three potential miR-184 target sites was cloned into the firefly luciferase vector (*Ncor2*-wt). For *Ncor2*-mut, six nt (underlined and in yellow) of the overlapping complementary sites were mutated. (C) Quantitative RT-PCR showed the increased levels of miR-184 upon overexpression. (D) Dual-luciferase reporter assay. *Ncor2*-wt construct in the presence of 10 pmol of miR-184 mimic showed the inhibitory activity of this reporter. The *Ncor2*-mut construct showed that miR-184 mimic cannot inhibit the luciferase activity compared with the wild-type construct. This experiment was repeated three times in triplicate. Values are presented as the mean ± SD. ***P < 0.001. (E) Western-blot assay. NCOR2 protein levels decreased in Hela cells after overexpression of miR-184 mimic (lane 184-mim) for 48 h compared with the cells transfected with scrambled control oligonucleotide (lane scramble). Arabic numbers demonstrate the NCOR2 average gray value normalized to β-Actin average gray value. (F) Quantitative RT-PCR showed *Ncor2 *mRNA did not change after overexpression of miR-184 in Hela cells compared with the negative control.

MiRNAs have been predicted to regulate genes expression through binding to the 3'UTR of the target mRNAs. Which gene might be the target of miR-184? To answer this question, TargetScan software [13] was used to screen the potential targets of miR-184. There are 18 predicted targets of miR-184 based on TargetScan screening. We were interested in *Ncor2*, because it has been reported that *Ncor2 *was expressed in the testis [14]. Our data also showed that the expression levels of *Ncor2 *mRNA and NCOR2 protein were higher in premature mouse testis than those in adult mouse testis (Figure 3), which showed an anti-correlation compared with the expression levels of miR-184 in developing mouse testis. Moreover, the 3'UTR of *Ncor2 *contains critical binding site (TCCGTCC) of miR-184. This sequence in the 3'UTR of *Ncor2 *is conserved among human, monkey, rat and mouse. Interestingly, there are three or two overlapping binding sites in the 3'UTR of *Ncor2 *in mouse or rat, respectively (Figure 4A). In addition, it has been shown that NCOR2 can interact with B-Myb to repress its transcription and thus inhibit cell proliferation [15-17]. Our data showed that overexpression of miR-184 in GC-1spg could promote its proliferation, suggesting that miR-184 might target *Ncor2 *to decrease its expression and reduce the amount of NCOR2 available for interaction with B-Myb. So the transcription level of B-Myb was increased, which may explain the proliferation-promoting effects of miR-184. Based upon the above-mentioned reasons, we focused our efforts on miR-184 and *Ncor2 *mRNA interactions.

To test whether miR-184 can alter the expression of *Ncor2*, we cloned the 3'UTR of *Ncor2 *mRNA containing the overlapping miR-184 binding sequences into a firefly luciferase reporter vector *(Ncor2*-wt) and cotransfected *Ncor2*-wt and a Rellina luciferase reporter vector and miR-184 into Hela cells, in which endogenous miR-184 was at very low or close to none levels (data not shown). As negative control, a scrambled oligonucleotide was assayed at the same time. The increased levels of miR-184 upon overexpression were demonstrated by quantitative RT-PCR (Figure 4C). By quantifying levels of the normalized luciferase activities in the presence of miR-184, we observed an ~ 45.8% decrease in luciferase activity with the construct bearing the 3'UTR of *Ncor2 *mRNA (Figure 4D). No decreases in luciferase activity was seen when miR-184 was replaced with a scrambled miRNA.

To investigate the specificity of interactions between miR-184 and *Ncor2 *target mRNA sequence, we created one mutation construct in the *Ncor2 *binding site for miR-184 (*Ncor2*-mut). In this construct, six nt of the overlapping binding site for miR-184 were mutated (Figure 4B). The *Ncor2*-mut did not decrease luciferase activity (Figure 4D).

To determine whether *Ncor2 *function as a target of miR-184 *in vivo*, Western-blot assays were used to test the protein levels of NCOR2 after overexpressed miR-184. As shown in Figure 4E, after transfected with miR-184-mim for 48 h, the NCOR2 protein levels in Hela cells were decreased compared with Hela cells transfected with scramble oligonucleotide. (Hela cells express endogenous NCOR2).

Taken together, we conclude that *Ncor2 *is the target gene of miR-184.

### MiR-184 downregulated *Ncor2 *through inhibiting NCOR2 protein translation *in vitro*

**Figure 5:**
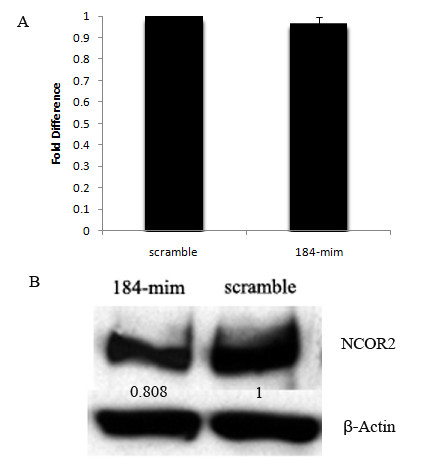
**MiR-184 downregulated *Ncor2 *expression through inhibiting NCOR2 protein translation in GC-1spg**. A, Quantitative RT-PCR results showed the levels of *Ncor2 *mRNA did not change after GC-1spg overexpression of miR-184. B, Western-blot results showed the expression levels of NCOR2 protein dropped after GC-1spg overexpression of miR-184. Arabic numbers demonstrate the NCOR2 average gray value normalized to β-Actin average gray value.

MiRNAs regulate gene expression at posttranscriptional levels by either preventing mRNAs from being translated or causing them to be degraded. To determine the mechanism of miR-184 downregulation of *Ncor2*, quantitative RT-PCR and Western-blot assays were performed. As shown in Figure 5A, the normalized mRNA levels of *Ncor2 *did not change after overexpression of miR-184 in GC-1spg compared with the scramble control. However, the NCOR2 protein levels normalized to β-Actin decreased (Figure 5B). Similar results were also demonstrated in Hela cells (Figure 4E, F). These results indicate that miR-184 could downregulate *Ncor2 *through inhibiting translation *in vitro*.

## Discussion

MiRNAs are small non-coding RNAs that play important roles in regulating post-transcriptional fate of mRNAs. Hundreds of miRNAs have been identified in plants and animals. About 68% of miRNAs are expressed in a highly tissue-specific manner [18]. Based on their tissue-specific/preferential and developmental expression patterns, miRNAs are believed to play prominent regulatory roles during development.

Previous studies have tested that miR-184 is only expressed in brain, testis and cortical epithelium in mouse [9,10]. Additionally, miR-184 expression levels in the testis were much higher than those in the brain [9]. Here, we found that miR-184 was expressed during the postnatal development of the mouse testis and its expression levels were increased with age (Figure 1). And we also demonstrated that miR-184 was localized to the germ cells of mouse testis from spermatogonia to round spermatids (Figure 1). As we known, a striking feature of spermatogenesis is that the gene expression patterns of spermatogenic cells exhibit spatiotemporal characteristics [19]. Taken together, the testis-preferential spatiotemporal expression pattern of miR-184 during postnatal development of the mouse testis indicates that miR-184 could play a role in mouse spermatogenesis.

Based on our finding that miR-184 was localized to germ cells of the mouse testis, we chose a germ cell line, GC-1spg, to identify the biological characteristics of miR-184 *in vitro*. Our data showed that overexpression of miR-184 in GC-1spg could induce GC-1spg cell cycle promotion and proliferation (Figure 2), which was similar to or different from the reports of other groups. Wong *et al *found that inhibition of miR-184 in tongue squamous cell carcinoma (SCC) cell lines could reduce cell proliferation rate, which indicates that miR-184 might play an oncogenic role in the antiapoptotic and proliferative processes of tongue SCC [20]. Chen and Forley *et al *found that miR-184 overexpression induced neuroblastoma cell cycle arrest and apoptosis through targeting the serine/threonine kinase AKT2 [21,22]. The discrepancy could be explained by the different targets of miR-184 in the different cell lines among the above investigations.

The prediction of mRNA targets for miRNAs will help characterize the miRNAs involved in biological processes and the molecular mechanisms via which miRNAs act. In this study, we have found that *Ncor2 *is a target gene of miR-184 (Figure 4). NCOR2 (also known as SMRT, silencing mediator for retinoic acid and thyroid hormone receptors) acts as a corepressor for a range of transcription factors including retinoic acid receptors (RARs), the retinoid X receptors (RXRs) and the thyroid hormone receptors (TRs) etc. In the absence of hormone ligand, NCOR2 binds to the unliganded nuclear receptors and serves as a platform for recruitment of additional components of a larger corepressor complex that includes histone deacetylase to inhibit gene transcription. And in the presence of hormone ligand, NCOR2 releases from the nuclear receptors, resulting the corepressor complex disruption and de-repression of gene expression [23-25]. Unlike the expression pattern of miR-184, *Ncor2 *is expressed ubiquitously. It is expressed in heart, liver, spleen, lung, kidney, skeletal muscle, intestine, brain and testis [17,26,27]. And the expression levels in brain are higher than those in testis [17], which is also different from the miR-184 expression levels between these two organs. Interesting, we also found that the expression levels of NCOR2 were decreased from postnatal 30 day testis to adult mouse testis comparing with those in postnatal 7 day testis (Figure 3), which was also different from our data showing that miR-184 expression levels increased during the postnatal development of mouse testis (Figure 1). The inverse expression patterns between miR-184 and NCOR2 are consistent with our data showing that miR-184 could downregulate *Ncor2 *through targeting its 3'UTR (Figure 4).

The downregulation of gene expression by miRNAs is a complex process involving both translational repression and mRNA degradation [2,28]. In this study, we have found that the mRNA of *Ncor2 *did not change after overexpression of miR-184 in GC-1spg and Hela cells. However, the NCOR2 protein levels did decrease after miR-184 overexpression in these two cell lines, indicating that miR-184 decreased NCOR2 protein levels not by mRNA degradation, but by translational repression *in vitro*. The mechanisms underlying translational repression by miRNAs include inhibition of translation initiation by competition between RISC (RNA induced silencing complex) and eIF4E (eukaryotic translation initiation factor 4E) for cap binding or by impeding the association of the small and large ribosomal subunits, inhibition of translation elongation and cotranslational degradation of nascent polypeptides [29]. The mechanism of how miR-184 regulates *Ncor2 *expression remains unresolved.

miR-184 was first observed in the murine eye [30]. Later, its expression pattern was determined by two groups [9,10]. About the biological functions of miR-184, studies have linked its overexpression to squamous cell carcinoma of the tongue [20], its downexpression to gliomas [31] and MYCN-amplified neuoblastoma [21,22]. Moreover, it has been reported that miR-184 has multiple roles in *Drosophila *female germline development [32]. In this study, we have found that the miR-184 expression levels were increased during the postnatal development of the mouse testis, miR-184 localization was restricted to testicular germ cells from spermatogonia to round spermatids, overexpression of miR-184 could promote GC-1spg proliferation and miR-184 downregulated *Ncor2 *by targeting its 3'-UTR through inhibiting NCOR2 protein translation.

## Conclusions

Based on our data, it is suggested that miR-184 may be involved in the post-transcription regulation of mRNAs such as *Ncor2 *in mammalian spermatogenesis.

## Methods

### Animals

The male C57BL/6J mice were used and the cares of animals were complied with the regulations of Shanghai Jiaotong University School of Medicine. Shanghai Jiaotong University School of Medicine approved this study. Testes were removed and immediately put into liquid nitrogen and then store at -80°C until ready to proceed with RNA or protein isolation or section for *in-situ *hybridization assay.

### RNA isolation, Reverse transcription and real-time PCR

Total RNA was extracted from mouse testes of postnatal day 7, 12, 17, 20, 30, 70 and from GC-1spg or Hela cells after transfected with miRNA mimics for 48 h using Trizol reagent (Invitrogen, Carlsbad, CA, USA) according to the instruction of the manufacturer. For *Ncor2*, cDNA was synthesized using the PrimeScript RT-PCR Kit (TaKaRa, Otsu, Shiga, Japan) and Quantitative real-time PCR was performed using the SYBR Premix Ex Taq (TaKaRa). *β-Actin *was used as an internal control for normalization. Primers for q-PCR were as followings: *Ncor2*: 5'-ACTGCCGCCCCTAAACGCAC-3' (sense), 5'-GGACCTCGGGATGCCTTGCG-3' (antisense). *β-Actin*: 5'- CAGCCTTCCTTCTTGGG-3' (sense), 5'-GGCATAGAGGTCTTTACGG-3' (antisense). For miR-184 and U6 snRNA, cDNA was synthesized with Bulge-loop primers and the Takara primeScript RT-PCT Kit (TaKaRa). U6 snRNA was used for normalization. Real-time PCR was done by using the SYBR Premix Ex Taq (TaKaRa). All quantitative real-time PCR was performed on an Applied Biosystems 7500 Real-time PCR system (Applied Biosystems, Carlsbad, CA, USA). The Bulge-loop primers and qPCR primers for miRNA-184 and U6 snRNA were synthesized from Ribobio (Guangzhou, China). The comparative threshold cycle method was used to calculate the relative gene expression.

### *In-situ *hybridization (ISH)

The method of *in-situ *hybridization assay was performed according to the procedure as described in Silahtaroglu *et al *[33] with minor modifications. Briefly, adult mouse testes were embedded in optimal cutting temperature (OCT) compound (Sakura Finetek, Torrance, CA, USA) and cut at 12 μm thickness. Tissues were fixed with 4% (wt/vol) paraformaldehyde in 0.1% DEPC-PBS for 10 min at room temperature, washed twice in DEPC-PBS, and were prehybridized for 30 min at 52°C in hybridization buffer [50% (vol/vol) formamide (Sigma-Aldrich, St. Louis, MO, USA), 5 × SSC (Ambion, Austin, TX, USA), 500 μg/ml yeast tRNA (Invitrogen, Carlsbad, CA, USA), 1 × Denhard's solution (Sigma-Aldrich, St. Louis, MO, USA) and DEPC treated water]. Tissues were hybridized in the presence of 2.5 pmol locked nucleic acid (LNA)-modified, digoxigenin (DIG)-labeled miR-184 probe (5'-DIG-ACCCTTATCAGTTCTCCGTCCA-3', Exiqon, Vedbaek, Denmark) for 4 h at 52°C. For negative control, an LNA-modified, DIG-labeled scramble probe (Exiqon, Vedbaek, Denmark) was used. Slides were washed in 0.1 × SSC at 61°C for three times followed by a wash in 2 × SSC at room temperature for 5 min. Immunological detection was carried out with the hybridization detection kit (Boshide, Wuhan, China) according to the manufacturer's manual. In brief, sections were treated with 3% H_2_O_2 _to block endogenous peroxidase and then washed with PBS for three times. After treatment with the blocking buffer, biotin labeled anti-digoxigenin antibody was added. After washes with PBS, sections were treated with streptavidin-biotin-peroxidase complex (SABC-POD), and biotin-labeled peroxidase was added. Following the final wash, diamido-benzidine (DAB) was used to detect the hybridization signal. Sections were then dehydrated, cleared, mounted, and imagined. Hematoxylin was used for cell nuclear staining.

### Plasmid constructs and miRNA mimics

*Ncor2*-wt: A 517 bp region of the 3'-UTR of mouse *Ncor2 *containing the overlapping potential miR-184 target sites was inserted into the *XbaI-FseI *site immediately downstream of the stop codon in the pGL3 Firefly Luciferase reporter vector (Promega, Madison, WI, USA). *Ncor2*-mut of the *Ncor2 *sequence was created by using a QuikChange Lightning Site-Directed Mutagenesis Kit (Stratagene, Santa Clara, CA, USA). miRNA mimics for miR-184 and cy3-labeled nontargeting scramble control were obtained from Ambion (Austin, TX, USA).

### Cell cycle assay

GC-1spg cells were cultured in Dulbecco modified Eagle medium (DMEM) with 10% fetal calf serum (FCS) and were seeded onto 6-well plate the day before transfection was performed. Cells (about 50% confluent) were transfected with miRNA mimics (100 pmol per well) with Lipofectamine 2000 (Invitrogen, Carlsbad, CA, USA). Forty eight hours after transfection, cells were harvested and fixed with 70% alcohol in D-PBS at 4°C. After washing in PBS, cells were treated with 1% RNase (w/v) at 37°C for 30 min. Cells were then stained with propidium iodide (250 μg/μl) at room temperature for 10 min and analyzed with a Flow cytometer (BD Biosciences, Sparks, MD, USA).

### Cell proliferation assay

GC-1spg cells were plated onto 24-well plate with triplicate wells for each transfection the day before transfection. Cells (about 40% confluent) were transfected with miRNA mimics (20 pmol per well) with Lipofectamine 2000 (Invitrogen, Carlsbad, CA, USA). After transfection for 0 h, 24 h, 48 h, 72 h, cells were treated with Cell Proliferation MTS Assay Kit (GENMED, Shanghai, China) [3-(4, 5-dimethylthiazol-2-yl)-5-(3-carboxymethoxyphenyl)-2-(4-sulfophenyl)-2H-tetrazolium, inner salt; MTS] according to the manufacture's manual. Briefly, cells were treated with 50 μl working solution per well under dim light. After culturing for 1 h at 37°C with 5% CO_2_, the culture media were transported into a 96-well plate and measured the absorbance at 490 nm using a microplate reader.

### Luciferase assay

Hela cells were cultured in DMEM with 10% FCS. On the day before transfection, cells were seeded onto 24-well plates. The next day, cells (about 80% confluent) were transfected with *NCOR2*-wt or *NCOR2*-mut (200 ng per well), pRL-TK control vector (coding for *Renilla *luciferase) (50 ng per well) and miRNA mimics (10 pmol per well). All transfections were carried out in triplicate with Lipofectamine 2000 (Invitrogen, Carlsbad, CA, USA). Twenty four hours after transfection, *Firefly *and *Renilla *luciferase activities were measured using the Dual Luciferase Reporter Assay (Promega, Madison, WI, USA).

### Western-blot

Hela cells and GC-1spg were seeded onto 24-well plates the day before transfections were performed. Cells (about 40% confluent) were transfected with miRNA mimics (20 pmol per well) with Lipofectamine 2000 (Invitrogen, Carlsbad, CA, USA). Forty eight hours after transfection, cell lysates were prepared with Cell LysisBuffer for Western and IP (Beyotime, Haimen, China). Proteins of postnatal 7, 12, 17, 20, 30 and 70 day testes were prepared with RIPA (Radio-Immunoprecipitation Assay) Lysis Buffer (Beyotime, Haimen, China). The concentrations of the proteins were determined using abicinchoninic acid protein assay kit (Pierce, Rockford, IL, USA). Samples were thawed in 5 × SDS-PAGE sample loading buffer, vortexed and then denatured at 100°C for 5 min and placed on ice for 5 min. Proteins were loaded in each lane 25 μg per lane and separated by SDS-PAGE. Resolving gels were cast using 6% acrylamide; stacking gels contained 5% acrylamide. Gels were equilibrated in TBS with Tween (TBST) and transferred to polyvinylidene fluoride (PVDF) membrane (Millipore, Billerica, MA, USA) by Wet Electrophoretic Transfer (Bio-Rad Laboratories). Blots were blocked in 5% nonfat milk in TBST at room temperature for 1 h and incubated with Rabbit anti-NCOR2 antibody (Abcam, Cambridge, MA, USA) diluted 1:200 or anti-β-Actin monoclonal antibody diluted 1: 2, 500 in TBST plus 5% nonfat milk overnight at 4°C with agitation. After complete washing in TBST, blots were incubated with anti-rabbit horseradish peroxidase conjugated IgG (diluted 1:10 000) or anti-mouse horseradish peroxidase conjugated IgG (diluted 1:10 000) at room temperature for 1 h, washed in TBST, and developed with ECL Plus reagents (Millipore, Billerica, MA, USA).

### Statistical analysis

A Student's *t *testing for comparison of two groups was used to analyze cell cycle change, cell proliferation assay and dual luciferase reporter assay. A *P *value of < 0.05 was considered statistically significant.

## Authors' contributions

JW participated in the design of the study, carried out the ISH assay, the cell cycle and MTS assay, miR-184 target test and Western-blot assay, drafted the manuscript. JB and LW participated in the real-time RT-PCR assay and Western-blot assay. YH participated in the preparation of plasmid constructs. CX conceived of the study, and participated in its design and coordination and helped draft the manuscript. All authors read and approved the final manuscript.

## Supplementary Material

Additional file 1 **The high transfection efficiency of cy3-labeled scramble control nucleotide in Hela cells and GC-1spg cells**. Under fluorescent light, red fluorescence could be observed in Hela cells and GC-1spg cells transfected with cy3-labeled scramble nucleotide by Lipofectamine 2000, indicating a high efficiency of transfection (more than 95% cells were transfected). A, B and C: Hela cells were transfected for 5 h, 24 h and 48 h, respectively. D: GC-1spg cells were transfected for 5 h. E, F, G and H are the same visions of A, B, C and D under normal white light, respectively. Click here for file
