# Supplementary figures and images for: MicroRNA-184 downregulates nuclear receptor corepressor 2 in mouse spermatogenesis
Source: BMC Dev Biol. 2011 Oct 24;11:64. doi: 10.1186/1471-213X-11-64 (PMC3227627; doi:10.1186/1471-213X-11-64)

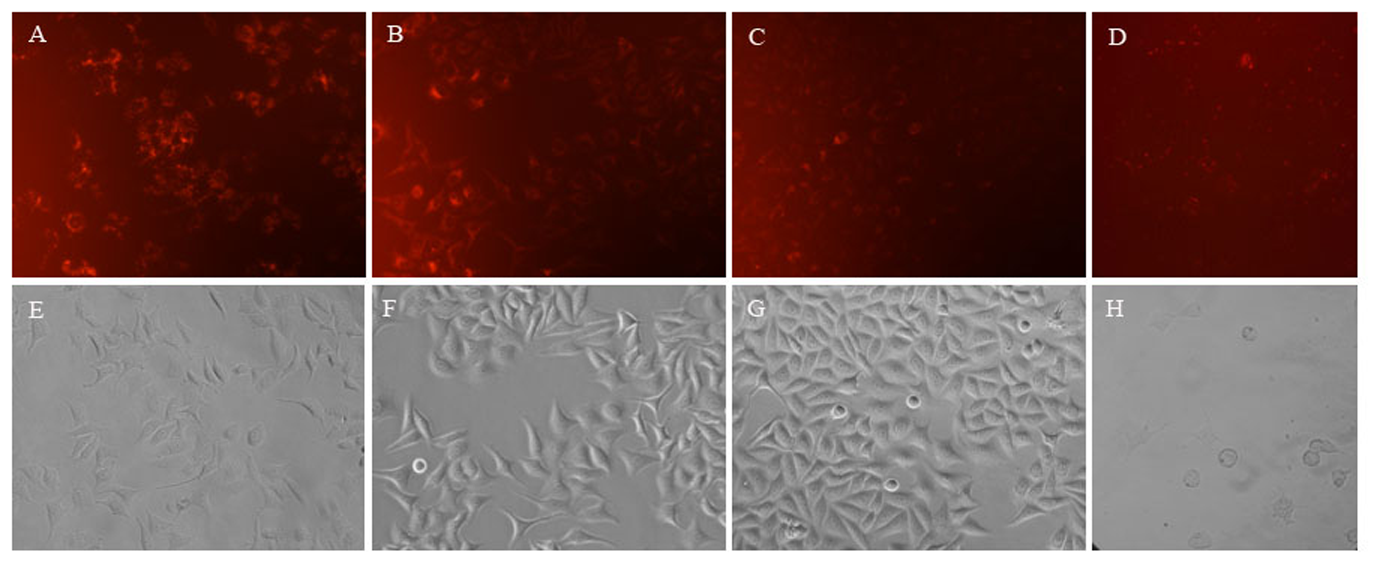

Supplement: Additional file 1 — The high transfection efficiency of cy3-labeled scramble control nucleotide in Hela cells and GC-1spg cells. Under fluorescent light, red fluorescence could be observed in Hela cells and GC-1spg cells transfected with cy3-labeled scramble nucleotide by Lipofectamine 2000, indicating a high efficiency of transfection (more than 95% cells were transfected). A, B and C: Hela cells were transfected for 5 h, 24 h and 48 h, respectively. D: GC-1spg cells were transfected for 5 h. E, F, G and H are the same visions of A, B, C and D under normal white light, respectively. [file 1471-213X-11-64-S1.TIFF]
